# Very High Blood Lead Levels Among Adults — United States, 2002–2011

**Published:** 2013-11-29

**Authors:** Kathryn Kirschner, Kathy Leinenkugel, Mike Makowski, Alicia M. Fletcher, Carol R. Braun, Walter A. Alarcon, Marie H. Sweeney, Geoffrey M. Calvert

**Affiliations:** Indiana Univ; Div of Environmental Health, Iowa Dept of Public Health; Div of Environmental Health Epidemiology, Pennsylvania Dept of Health; Bur of Occupational Health and Injury Prevention, New York State Dept of Health; Bur of Environmental Epidemiology, Missouri Dept of Health and Senior Svcs; Div of Surveillance, Hazard Evaluations, and Field Studies, National Institute for Occupational Safety and Health, CDC

Over the past several decades there has been a remarkable reduction in environmental sources of lead, improved protection from occupational lead exposure, and an overall decreasing trend in the prevalence of elevated blood lead levels (BLLs) in U.S. adults. As a result, the U.S. national BLL geometric mean among adults was 1.2 *μ*g/dL during 2009–2010 (1). Nonetheless, lead exposures continue to occur at unacceptable levels (2). Current research continues to find that BLLs previously considered harmless can have harmful effects in adults, such as decreased renal function and increased risk for hypertension and essential tremor at BLLs <10 *μ*g/dL (3–5). CDC has designated 10 *μ*g/dL as the reference BLL for adults; levels ≥10 *μ*g/dL are considered elevated (2). CDC’s Adult Blood Lead Epidemiology and Surveillance (ABLES) program tracks elevated BLLs among adults in the United States (2). In contrast to the CDC reference level, prevailing Occupational Safety and Health Administration (OSHA) lead standards allow workers removed from lead exposure to return to lead work when their BLL falls below 40 *μ*g/dL (6). During 2002–2011, ABLES identified 11,536 adults with very high BLLs (≥40 *μ*g/dL). Persistent very high BLLs (≥40 *μ*g/dL in ≥2 years) were found among 2,210 (19%) of these adults. Occupational exposures accounted for 7,076 adults with very high BLLs (91% of adults with known exposure source) and 1,496 adults with persistent very high BLLs. Adverse health effects associated with very high BLLs (4,5,7) underscore the need for increased efforts to prevent lead exposure at workplaces and in communities.

Forty-one states participated in the ABLES program in 2011.* These states received adult BLL data from laboratories and physicians through mandatory reporting. Adults were defined as persons aged ≥16 years at the time of BLL testing. Each state ABLES program assigned a unique identifier to each adult to protect individual privacy while permitting longitudinal analyses (2). For this analysis, a BLL ≥40 *μ*g/dL was defined as a very high BLL. A very high BLL measured over a period ≥2 years was defined as a persistent very high BLL. The number of adults with very high BLLs and the number with persistent very high BLLs during 2002–2011 were counted. Persistent very high BLLs can result in spontaneous abortion, reduced newborn birthweight, neurocognitive deficits, sperm abnormalities, subclinical peripheral neuropathy, hypertension, anemia, kidney dysfunction, and nonspecific symptoms (4,5). As part of their regular activities, and to the extent resources allow, state ABLES programs 1) investigate the circumstances associated with reports of elevated BLLs; 2) contact health-care providers, workers, and employers to gather industry and occupation data and additional exposure information and provide information and educational materials; and 3) refer employers in occupational cases to OSHA offices for technical assistance or enforcement of the lead standards.

From 2002 to 2011, a total of 11,536 adults had very high BLLs among the 1,201,669 adults reported to the ABLES program during this period. Among these adults, 2,210 (19%) had persistent very high BLLs, 1,487 (13%) had BLLs ≥60 *μ*g/dL, and 96 had BLLs ≥60 *μ*g/dL for ≥2 years (Table 1). A total of 7,076 adults with very high BLLs (91% of adults with known exposure source) were exposed at work (Table 2), and 1,496 of these had persistent very high BLLs (93% of adults with known exposure source). These 7,076 workers were predominantly employed in the manufacturing, construction, services, or mining sectors. Within this group, 49% of the workers were employed in three subsectors (i.e., battery manufacturing, nonferrous metal production and processing, and painting and wall covering contractors). Shooting firearms; remodeling, renovating, or painting; and using lead-containing alternative medicines (Table 2) were the most common sources for nonoccupational very high BLLs. The following four case histories illustrate the persistent problem of adults with very high BLLs in the United States (Figure).

## Case Histories

### Worker A

Worker A is a man aged 48 years who was working for a bridge painting firm and was responsible for recycling grit and steel shot from sandblasting operations. His primary protection from lead exposure was an air-supplied sandblasting hood. This worker had BLLs of 67 *μ*g/dL in May and June of 2010. He was removed from all work and received chelation treatments (therapy to remove heavy metals from the body). His BLL dropped to 42 *μ*g/dL in July and to 26 *μ*g/dL in September 2010. At last contact in February 2011, he continued to be removed from work per physician orders.

### Worker B

Worker B is a painter for a small construction company, aged 46 years, who, in December 2007, was seen in a hospital emergency department because of severe stomach pain. His BLL was 143 *μ*g/dL; he was given a chelation treatment and was followed at an occupational health clinic. His last BLL on record in February 2010 was 13 *μ*g/dL. At the time of initial testing, this worker was scraping paint from a house that was >100 years old. He used no respirator and wore no protective clothing except for gloves. He was not informed about lead hazards. His employer did not provide laundry services or disposable clothing. No other source of lead exposure was identified.

### Worker C

Worker C is a man aged 45 years who began working for a battery manufacturing company in May 2000. He worked in maintenance and was responsible for cleaning under lead pots. He did not use a respirator as instructed by his employer because of the heat in the factory. His first BLL was 25 *μ*g/dL in March 2001, and by August 2002 his BLL was 60 *μ*g/dL. Because of the very high BLL, the company moved him into a job with low likelihood of lead exposure. After his BLL dropped below 40 *μ*g/dL in November 2002, he was reassigned to job duties with high likelihood of lead exposure. When his BLL rose to 40 *μ*g/dL again in April 2003, the company issued him a full-face respirator and required him to use it. He was also instructed to shower at the end of the day and for breaks and lunch. He later transferred into jobs at the plant with lower lead exposures and his BLL continued to drop. His most recent BLL in August 2011 was 14 *μ*g/dL.

### Adult D

Adult D is a man aged 60 years with a BLL of 84 *μ*g/dL in March 2009 who had no known occupational exposure. His known exposures were target shooting at an indoor shooting range and casting bullets. He was given a chelation treatment in April 2009. Approximately 100 BLLs analyzed through June 2012 ranged from 6 *μ*g/dL to 84 *μ*g/dL, with a mean of 28 *μ*g/dL. He and his physician were informed about the detrimental effects of lead and how to limit his exposure, but the patient continues his two hobbies.

### Editorial Note

Reducing lead exposures at work and in the community is essential to avoid adverse health effects in humans. Reducing by 10% the rate of persons who have elevated BLLs (i.e., BLLs ≥10 *μ*g/dL) from work exposures is a *Healthy People 2020* objective (OHS-7) (8). The 2010 baseline rate for BLLs ≥10 *μ*g/dL is 26.4 adults per 100,000 employed adults (2). Reducing adverse health effects resulting from lead exposures requires 1) adherence to engineering controls and safe work practices; 2) BLL testing and management of elevated BLLs according to the most current medical guidelines and recommendations; and 3) education in the workplace and community (4–7,9). OSHA lead standards give the examining physician broad flexibility to tailor special protective procedures to the needs of individual employees (6). Therefore, the most current guidelines for management of lead-exposed adults (4,5,7) should be implemented by the medical community at the current CDC reference BLL of 10 *μ*g/dL (2), including consideration of removal from lead exposure at lower levels than the current OSHA lead standards require. Increasing the number and timeliness of referrals to OSHA of workplaces identified by state ABLES programs fosters prompt intervention and mitigation of lead exposure hazards.

What is already known on this topic?

The vast majority of elevated blood lead levels (BLLs) in the United States are workplace-related. Most lead exposures at work occur in the manufacturing, construction, services, and mining industries. Current research has found that even BLLs <10 *μ*g/dL can cause harm in adults. CDC considers BLLs ≥10 *μ*g/dL to be elevated. In contrast to the CDC reference level, prevailing Occupational Safety and Health Administration (OSHA) lead standards allow workers removed from lead exposure to return to lead work when their BLL falls below 40 *μ*g/dL.

What is added by this report?

Data collected by the Adult Blood Lead Epidemiology and Surveillance program during 2002–2011 identified 11,536 adults with very high BLLs (≥40 *μ*g/dL), of whom 19% had elevated BLLs recorded during ≥2 years. Among those with known exposure source, occupational exposures accounted for 91% of adults with very high BLLs.

What are the implications for public health practice?

The finding that many workers have harmful BLLs, some that are persistent for ≥2 years, is of grave concern. Examining physicians should be aware that the OSHA lead standards give them broad flexibility to tailor protections to the worker’s needs, including consideration of removal from lead exposure at BLLs lower than the current OSHA lead standards require. To prevent adverse health outcomes caused by very high BLLs, public health practitioners need to increase lead exposure prevention activities directed at employers, workers, health-care providers, and the community.

The findings of this report demonstrate that many adults in the United States continue to have very high BLLs. The fact that some adults had persistent very high BLLs is of grave concern. These adults were chronically exposed to lead above BLLs known to cause neurologic, cardiovascular, reproductive, hematologic, and kidney adverse effects (3–5). The risks for adverse chronic health effects are even higher if the exposure is maintained for many years (4,5,7).

The findings in this report are subject to at least three limitations, all of which suggest that ABLES underestimates the number of adults with elevated BLLs. First, employers might not provide BLL testing to all lead-exposed workers as required by OSHA regulations (10). Second, nonoccupationally exposed adults might not be tested. Finally, some laboratories might not report all tests as required by state laws or regulations (Susan Payne, California Department of Public Health, personal communication, June 18, 2012).

Possible factors contributing to the persistence of very high BLLs include 1) prevailing OSHA lead standards require medical removal from lead exposures only after a construction worker’s BLL reaches or exceeds 50 *μ*g/dL or a general industry worker’s BLL reaches or exceeds 60 *μ*g/dL; 2) examining physicians rarely recommend more stringent worker protections, which the OSHA lead standards allow but do not require; 3) some employers fail to implement appropriate engineering protections and workplace controls; 4) some adults fail to comply with safe practices and behaviors; and 5) state ABLES programs do not always have the resources to investigate and refer to OSHA all cases with very high BLLs.

Very high BLLs continue to be documented in adults in the United States. Actions that might decrease the number of adults with harmful BLLs include 1) increased employer efforts to reduce work-related lead exposure (6,9) and to comply with current guidance (4,7) for testing and managing lead-exposed workers; 2) adherence by lead-exposed workers to safe work practices, such as properly using personal protective equipment, washing before eating, and showering and changing clothes before going home; 3) education of the medical community to use current guidelines and recommendations for management of lead-exposed adults with BLLs ≥10 *μ*g/dL (4,5,7); and 4) increased involvement of the public health community to prevent nonoccupational lead exposures.

## Figures and Tables

**FIGURE:**
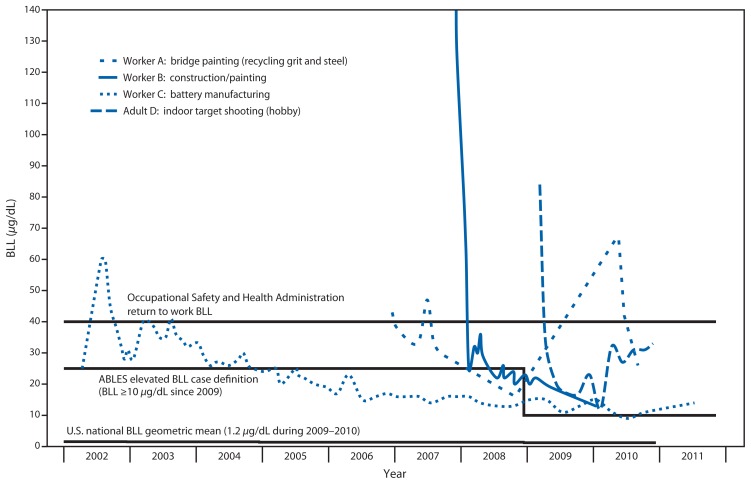
Four adults with very high blood lead levels (BLL ≥40 *μ*g/dL) in multiple years, by year — Adult Blood Lead Epidemiology and Surveillance (ABLES) Program, United States, 2002–2011

**TABLE 1:** Number of adults with very high blood lead levels (BLLs ≥40 *μ*g/dL) in multiple years — Adult Blood Lead Epidemiology and Surveillance (ABLES) Program, United States, 2002–2011

Characteristic	No. of adults with BLLs ≥40 *μ*g/dL	No. of adults with BLLs ≥60 *μ*g/dL*
No. of years with very high BLLs		
1	9,326	1,391
2	1,415	74
3	402	17
4	197	2
5	91	2
6	50	0
7	29	1
8	8	0
9	13	0
10	5	0
Total no. of adults with at least one very high BLL in 10 years	11,536	1,487
Total no. of adults with persistent very high BLLs (≥2 years)	2,210	96

* Adults with BLLs ≥60 *μ*g/dL are a subset of those adults with BLLs ≥40 *μ*g/dL.

**TABLE 2:** Number and percentage of adults with very high blood lead levels (BLLs ≥40 *μ*g/dL), by industry subsector, and number and percentage by nonoccupational sources of exposure — Adult Blood Lead Epidemiology and Surveillance (ABLES) Program, United States, 2002–2011

	40–59 *μ*g/dL		≥60 *μ*g/dL		Total (≥40 *μ*g/dL)	
			
Characteristic	No.	(%)	No.	(%)	No.	(%)
**Overall**	**10,049**	**(100.0)**	**1,487**	**(100.0)**	**11,536**	**(100.0)**
**Exposure type**						
Occupational	6,330	(63.0)	746	(50.2)	7,076	(61.3)
Nonoccupational	497	(4.9)	166	(11.2)	663	(5.7)
Unknown exposure source	3,222	(32.1)	575	(38.7)	3,797	(32.9)
**Industry subsector [NAICS codes]**	**6,330**		**746**		**7,076**	**(100.0)**
**Manufacturing**	**3,393**	**(100.0)**	**253**	**(100.0)**	**3,646**	**(51.5)**
Battery manufacturing [33591]	1,671	(49.2)	70	(27.7)	1,741	
Nonferrous metal production and processing [3313 and 3314]	577	(17.0)	47	(18.6)	624	
Foundries [3315]	244	(7.2)	28	(11.1)	272	
Fabricated metal product manufacturing [332]	257	(7.6)	31	(12.3)	288	
Other manufacturing industries	644	(19.0)	77	(30.4)	721	
**Construction**	**1,575**	**(100.0)**	**318**	**(100.0)**	**1,893**	**(26.8)**
Painting and wall covering contractors [23832]	890	(56.5)	185	(58.2)	1,075	
Highway, street, and bridge construction [23731]	262	(16.6)	37	(11.6)	299	
Site preparation contractors [23891]	96	(6.1)	21	(6.6)	117	
Other construction industries	327	(20.8)	75	(23.6)	402	
**Services (except public safety)**	**555**	**(100.0)**	**87**	**(100.0)**	**642**	**(9.1)**
Remediation services [56291]	186	(33.5)	27	(31.0)	213	
All other amusement and recreation industries [71399]	102	(18.4)	17	(19.5)	119	
Automotive repair and maintenance [8111]	71	(12.8)	9	(10.3)	80	
Other services industries	196	(35.3)	34	(39.1)	230	
**Mining (except oil and gas extraction)**	**428**	**(100.0)**	**16**	**(100.0)**	**444**	**(6.3)**
Lead ore and zinc ore mining [212231]	418	(97.7)	14	(87.5)	432	
Other mining industries	10	(2.3)	2	(12.5)	12	
**Other/missing industry data**	**379**		**72**		**451**	**(6.4)**
**Nonoccupational exposures**	**497**	**(100.0)**	**166**	**(100.0)**	**663**	**(100.0)**
Shooting firearms (target shooting)	144	(29.0)	17	(10.2)	161	(24.3)
Remodeling/renovation/painting	89	(17.9)	22	(13.3)	111	(16.7)
Complementary and alternative medicines (e.g., Ayurvedic medicines)	31	(6.2)	28	(16.9)	59	(8.9)
Eating food containing lead	39	(7.8)	18	(10.8)	57	(8.6)
Retained bullets (gunshot wounds)	30	(6.0)	15	(9.0)	45	(6.8)
Casting (e.g., bullets and fishing weights)	33	(6.6)	6	(3.6)	39	(5.9)
Pica (i.e., the eating of nonfood items)	16	(3.2)	20	(12.0)	36	(5.4)
Other or unknown nonoccupational source	115	(23.1)	40	(24.1)	155	(23.4)

**Abbreviation:** NAICS = North American Industry Classification System.

## Footnotes

* Federal funding for state ABLES program was discontinued in September 2013. The ABLES program continues to provide technical assistance to states with adult blood lead surveillance programs and maintains the ABLES website for reporting ongoing analyses of ABLES data.
