# Errata

**Published:** 2014-01-03

## Vol. 62, No. SS-8

In the Surveillance Summary, “Abortion Surveillance — United States, 2010,” on page 19 in Table 2, under the column for abortions reported by residence, the following numbers, rates, and ratios of abortions should have been reported for Maine, Massachusetts, Pennsylvania, Rhode Island, Virginia, and Washington:

**Table:** 

	Residence		
	
State/Area	No.	Rate	Ratio
Maine	2,251	9.3	174
Massachusetts	20,398	15.1	280
Pennsylvania	37,738	15.5	263
Rhode Island	3,480	16.2	311
Virginia	25,906	15.7	252
Washington	21,096	15.6	244

## Vol. 62, No. 47

In the report, “Very High Blood Lead Levels Among Adults — United States, 2002–2011,” several errors occurred. On page 967, the ninth sentence of the first paragraph should read, “Persistent very high BLLs (≥40 *μ*g/dL in **>1 calendar year**) were found among 2,210 (19%) of these adults.” The sixth sentence of the second paragraph should read, “A very high BLL measured **in >1 calendar year** was defined as a persistent very high BLL.” The second sentence of the third paragraph should read, “Among these adults, 2,210 (19%) had persistent very high BLLs, 1,487 (13%) had BLLs ≥60 *μ*g/dL, and 96 had BLLs ≥60 *μ*g/dL **in >1 calendar year** (Table 1).” On page 968, the last row header of Table 1 should read, “Total no. of adults with persistent very high BLLs (**in >1 calendar year**).” On page 970, in the “What is added by this report?” section of the summary box text, the first sentence should read, “Data collected by the Adult Blood Lead Epidemiology and Surveillance program during 2002–2011 identified 11,536 adults with very high BLLs (≥40 *μ*g/dL), of whom 19% had elevated BLLs recorded **in >1 calendar year**.” Finally, in the “What are the implications for public health practice?” section of the summary box, the first sentence should read, “The finding that many workers have harmful BLLs, some that are **present for >1 calendar year**, is of grave concern.”

